# Detection and Characterization of Goose Astrovirus Infections in Hatcheries and Commercial Goose Flocks

**DOI:** 10.1155/2023/1127544

**Published:** 2023-02-27

**Authors:** Zhihao Ren, Qingshui Zhang, Jinxin Li, Ziding Yu, Guanghua Fu, Rongchang Liu, Yu Huang, Jingliang Su

**Affiliations:** ^1^Key Laboratory of Animal Epidemiology of the Ministry of Agriculture, College of Veterinary Medicine, China Agricultural University, Beijing 100193, China; ^2^Institute of Animal Husbandry and Veterinary Medicine, Fujian Academy of Agricultural Sciences, Fuzhou 350013, China

## Abstract

Goose astrovirus (GoAstV) has frequently been isolated in China since it was first identified as the etiological agent of visceral gout in goslings in 2017. However, the actual prevalence of GoAstV infection and its economic impact on commercial goose production remain poorly characterized. Here, virus detection and serological testing were conducted to determine the extent of GoAstV infection in commercial goose flocks. We detected GoAstV RNA in 2% (6/300) of dead-in-shell embryos and day-old hatched goslings by RT-PCR, indicating vertical transmission under natural conditions. Using a virus neutralization test, GoAstV antibodies were detected in 41.7%–61.1% of serum samples from four commercial goose flocks, indicating that infections were common. To determine the virus types circulating in the commercial flocks, we isolated 15 GoAstVs from goose tissue samples from farms located in five provinces during 2018–2022. Genomic sequence analysis showed that all sequences were corresponded to GoAstV group 2 (GoAstV-2) but were assigned into three capsid subgroups based on sequence variations in the capsid protein. Representative isolates of capsid subgroups were also antigenically evaluated using cross-neutralization tests in LMH cell cultures. The antigenic relatedness values (*R*) calculated using the Horsfall formula were between 62% and 86%, indicating that no significant antigenic differences exist between the isolates. Our findings indicate that GoAstV-2 viruses are an important cause of fatal gout in goose flocks, as well as hatchery contamination in China.

## 1. Introduction

Astroviruses (AstVs) are small, nonenveloped, single-stranded RNA viruses belonging to the *Astroviridae* family. They infect a variety of hosts from mammals to birds and are divided into two genera, *Mamastrovirus* (MAstV) and *Avastrovirus* (AAstV). AstVs have a genome of approximately 7 kb, including a 5′-untranslated region (UTR), three open reading frames (ORF1a, ORF1b, and ORF2), and a 3′-UTR with a poly(A) tail. ORF1a and ORF1b encode nonstructural proteins, and ORF2 encodes the viral capsid protein that encapsulates the viral genome [1].

In most species, AstVs are associated with gastroenteritis, but AAstV infections lead to more diverse disease phenotypes. For example, AAstVs have been documented to cause lethal hepatitis in domestic ducklings [2], nephritis or white chick in chickens, and poult enteritis mortality syndrome with high mortality [3–5]. AAstV infection has also been implicated in prehatching mortality and growth depression in poultry, leading to heavy economic losses in farms [6, 7]. In 2017, a fatal gout of goslings caused by a goose astrovirus (GoAstV) was first described [8]. Subsequent studies reported that the disease occurred in varying numbers of goose flocks throughout the Chinese mainland, typically affecting flocks at 1–3 weeks of age, with a mortality rate of 10%–30% [9–12]. Moreover, GoAstV infections in ducklings and chickens have been reported recently [13–15]. The emergence of GoAstV and its high pathogenicity imposes a great challenge to the health of geese and to the poultry industry generally. However, the nature and extent of the disease remain largely unclear. In this study, we reported the detection of GoAstV genes in dead-in-shell embryos and day-old hatched goslings, along with the prevalence of neutralizing antibodies in four commercial goose flocks. In addition, GoAstVs isolated from clinically dead goose samples were characterized.

## 2. Materials and Methods

### 2.1. Sample Origin and Processing

In this study, four cohorts of samples were tested, as described as follows:

- *Goose Embryos Cohort*. Dead-in-shell embryos in the final stage of incubation were collected from a hatchery at Jian, Jiangxi province, in 2019. The fertilized eggs came from breeding goose flocks of Farm A located in Chuzhou, Anhui province. After dissection, the kidney and liver tissues were individually collected with aseptic tools and homogenized with sterile PBS to a suspension of 20% (w/v).
- *Cloacal Swab Cohort*. Swabs were collected from day-old hatched goslings in a hatchery in Anhui province during three farm visits from 2019 to 2022. The fertilized eggs came from Farm A, as previously mentioned. Collected swabs were immersed individually in tubes containing 0.5 ml of prechilled Dulbecco's modified Eagle's medium (DMEM). After vigorous vortexing, the sample was centrifuged, and the aliquoted supernatants were stored at −75°C prior to use.
- *Dead Geese Cohort*. Dead geese from a disease outbreak were submitted for laboratory diagnosis from 2017 to 2022. The flocks were distributed throughout Hebei, Shandong, Jiangsu, Anhui, Jiangxi, and Guangdong provinces in China. At necropsy, the kidney, spleen, and liver tissues of the dead geese with gross lesions of visceral gout were collected.
- *Goose Serum Cohort*. The samples of goose serum used in this investigation were collected from two different sources. One set from a replacement breeder goose flock of 60 days old was in Farm A. Another three sets of serum samples were collected from *foie gras* geese during three visits to a slaughterhouse at Farm B in Weifang, Shandong province. This was a “closed-circuit” farm, in which 1-day-old goslings were introduced and reared in semiopen houses to 90 days old. Then, the geese were gavaged until 120–130 days of age. Sera collected at slaughtering were heat-inactivated at 56°C for 30 min before use.

### 2.2. RT-PCR Detection

The presence of AstV in samples from the embryo and cloacal swab cohorts was detected by hierarchical testing. Pools of 10 samples (10-in-1 test) were initially detected using RT-PCR, as previously described [16]. In brief, total RNA was extracted from pooled tissues or swab supernatants using a viral RNA kit (Omega, Bio-Tek, Norcross, GA), and cDNA was synthesized from RNA using a reverse transcription system (Promega, WI, USA) with random primers following the manufacturer's instructions. The presence of AstV cDNA was detected by heminested-PCR amplification with the pan-AstV degenerate primers targeting the viral RdRp gene [16]. The positive pools were returned and detected singly.

### 2.3. Virus Isolation and Genome Sequencing

To isolate GoAstV, the kidney, spleen, and liver tissues of clinically dead geese in each case were treated and inoculated into 10- to 12-day-old healthy goose embryos via the chorioallantoic membrane route, as described previously [8]. After three sequential passages, the infected allantoic fluid was harvested, and the presence of GoAstV was detected by RT-PCR.

The complete genomes of all isolates were amplified using eight pairs of primers and sequenced as previously reported [12]. Trees were constructed based on the RdRp nucleotide sequences and the amino acid sequences of ORF2 [17].

### 2.4. Virus and Cell Culture

Chicken liver hepatocellular carcinoma cells (LMH, ATCC) were maintained in a growth medium consisting of DMEM and F12 (1 : 1) containing 10% fetal bovine serum (FBS) and 100 U/ml of penicillin/streptomycin at 37°C with 5% CO_2_. Goose embryo propagated astrovirus AAstV/Goose/CHN/2017/SD01 (SD01) (GenBank accession no. MF772821) was inoculated onto an LMH cell monolayer with 80% confluence at a dilution of 1 : 10 in DMEM. After 2 h incubation, the supernatant was removed and replaced with DMEM/F12 containing 1 *μ*g/ml TPCK (L-1-tosylamido-2-phenylethyl chloromethyl ketone)-treated trypsin [18]. The infected cells were maintained and passaged every 5 days, and the viral antigen was detected by an indirect immunofluorescence assay and RT-PCR.

### 2.5. Generation of Monoclonal Antibody (MAb) F6B2

To produce MAb F6B2, the ORF2 gene was amplified using a primer pair (F: 5ʹ-AAAACTGCAGGTGGCGGACCGAAATAAAATGGCAGACAGGGCGGTGGCC CCGCGCGAG-3ʹ, R: 5ʹ-CCGCTCGAGTCAGTGATGATGATG ATGATGCTCATGT CCGCCCTTCTC-3ʹ) from the GoAstV SD01 genome and inserted into the pFastBac1 plasmid. The recombinant capsid protein was produced using the Sf9-Baculovirus system following a previously described protocol [19]. Eight-week-old BALB/c mice were subcutaneously immunized with 100 *μ*g of purified recombinant protein at a 1 : 1 ratio with Freund's complete adjuvant, followed by two additional subcutaneous boosters of the purified protein with Freund's incomplete adjuvant and one intraperitoneal injection without adjuvant at 2-week intervals. Mice were euthanized 3 days after the last immunization, and splenocytes were isolated and fused with SP2/0 cells. Hybridomas secreting capsid protein-specific antibodies were first screened using an immunofluorescence assay as described previously. The selected hybridoma was cloned by three rounds of limiting dilution and amplified as mouse ascitic fluid.

### 2.6. Immunofluorescence Assay

An indirect immunofluorescence assay (IFA) was performed using mouse ascitic monoclonal antibody MAb F6B2 as the primary antibody. Briefly, cells in 96-well plates were washed once with PBS and then fixed with a prechilled acetone/methanol (1 : 1) mixture for 20 min at room temperature. The fixed cells were washed three times with PBS and incubated at 37°C for 30 min with PBS containing 5% skim milk and 0.1% Tween-20. Next, wells were gently washed with PBS, and MAb F6B2 was added at a dilution of 1 : 2000 with PBS. After incubation at 37°C for 1 h, cells were washed three times with PBS, and the secondary antibody of DyLight488-conjugated goat antimouse IgG (Canlifesci Inc. Co. Ltd., Beijing, China) at a 1 : 800 dilution was added. The plate was incubated at room temperature for 1 h. Incubation with 1 *μ*g/ml DAPI stain was finally performed for 15 min, and then, the cells were washed three times with PBS. Uninfected cells were used as a negative control.

### 2.7. Serum Neutralization

A virus neutralization (VN) test to measure the antibodies in the serum samples was conducted on LMH cell cultures. We used GoAstV strain SD01 that was adapted in LMH cells for 20 passages. Initial screening for the presence of antibodies was conducted by making a single dilution of the tested sera at a 1 : 10 ratio with DMEM, and furthermore, twofold serial dilutions were made for the positive sera to determine antibody titers. When LMH cells grew to 80% confluence in 96-well plates, the diluted serum samples were individually mixed with an equal volume of SD01 working suspension containing 200 TCID_50_ in 0.1 ml. After incubation for 1 h at 37°C, the serum-virus mixture was added to five cell wells (0.1 ml/well). After incubation at 37°C for 1 h, the inoculum in each well was removed, and cells were washed once with PBS, and DMEM/F12 containing 1 *μ*g/ml TPCK-treated trypsin was added and incubated for 5 days. Then, residual AstV was detected in the cells by IFA as described previously. As a positive control, goose serum immunized with strain SD01 was incubated with the virus, and mock incubation of the virus was conducted. The 50% endpoint of neutralization (NT_50_) was calculated by the Reed–Muench method [20].

### 2.8. Cross-Reactivity Assay of AstV Isolates

To prepare antisera against AstV, four representative isolates, namely SD01, GD01 (GenBank accession no. OP484923), JX-Embryo (GenBank accession no. OP621333), and SD05 (GenBank accession no. OP020131), were selected. Groups of three antibody-negative adult geese were subcutaneously injected two times with a dose of 10^4^ ELD_50_ per goose at an interval of two weeks. Antisera were collected after the second injection, and heat inactivated for 30 min at 56°C.

A VN test to measure the cross-reactivity of these representative isolates was carried out using the microneutralization method performed on LMH cells as described previously [21] with some modifications. Briefly, GoAstV isolates were individually adapted in LMH cells by five serial passages. Serum neutralization was carried out against 200 TCID_50_ of each isolate, and the neutralization titers against homologous and heterologous sera were determined after a 5-day incubation period. To assess the antigenic relationship between the four isolates, the titer ratio “*R*” values were calculated using the homologous and heterologous serum titers (NT_50_) as follows [22]: *R* = $\sqrt{r1\times r2}$ × 100%, where *r*1 = heterologous titer (virus 2)/homologous titer (virus 1); *r*2 = heterologous titer (virus 1)/homologous titer (virus 2). An *R* value of 24%–49% represented a four- to eightfold difference, and 0%–24% indicated a greater than eightfold difference [23].

## 3. Results

### 3.1. Prevalence of GoAstV in Hatchery Embryos and Day-Old Goslings

Using the pan-AstV RT-PCR, AstV RNA was detected from the kidney/liver tissues in six of the 300 dead-in-shell embryos (positive rate = 2%). We next investigated the presence of AstV in day-old hatched goslings by testing cloacal swab samples. AstV RNA was detected in 12 (6%), 8 (4%), and 10 (5%) of the three batches of samples (*n* = 200).

### 3.2. Serological Testing of Commercial Goose Flocks

**Figure 1:**
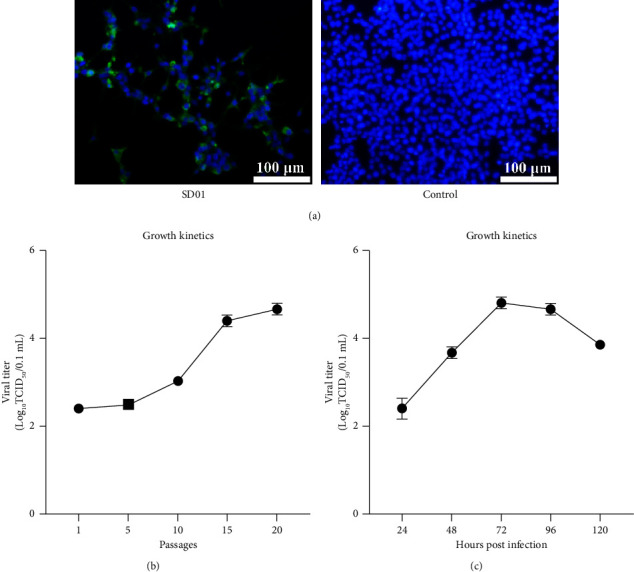
Detection and growth kinetics of LMH cells infected with astrovirus strain SD01. (a) Indirect immunofluorescence staining of GoAstV-infected LMH cells with monoclonal antibody F6B2. (b, c) Growth kinetics of strain SD01 in LMH cells at different passages and at passage 20 post-infection.

To develop a microplate assay for the assessment of neutralizing antibodies against GoAstV, the GoAstV isolate SD01 was first serially passaged in LMH cells. The adaptation of the virus and the presence of viral antigens in infected cells were confirmed by IFA with monoclonal antibodies (Figure 1(a)). Quantitative assay results indicated that the infectious virus titer increased with passaging and peaked at 72 h after infection with a titer of approximately 10^4.5^ TCID_50_/0.1 ml (Figures 1(b) & 1(c)). Using the virus SD01 that had been propagated in LMH cells, we developed a microneutralization test. We first tested 10 serum samples collected from convalescent geese in previous infection experiments, and all sera neutralized the virus at a dilution greater than 1 : 10. By contrast, the negative sera did not neutralize the virus at the initial dilution of 1 : 5. We therefore selected to test sera at a dilution of 1 : 10 to assess the presence of neutralizing antibodies. Using this test approach, all four goose flocks were seropositive for GoAstV, with the positive detection rate ranging from 41.7% to 61.1% (Table 1).

### 3.3. Virus Isolation and Molecular Characterization

By inoculating healthy goose embryos, we isolated 15 AstVs from the tissue samples of clinically dead geese from different regions, as indicated in Table 2. The SD05 strain was isolated from the tissues of geese that died at 50 days old, suggesting that pathogenic AstV persisted and caused fatal infection in older geese under field conditions.

**Figure 2:**
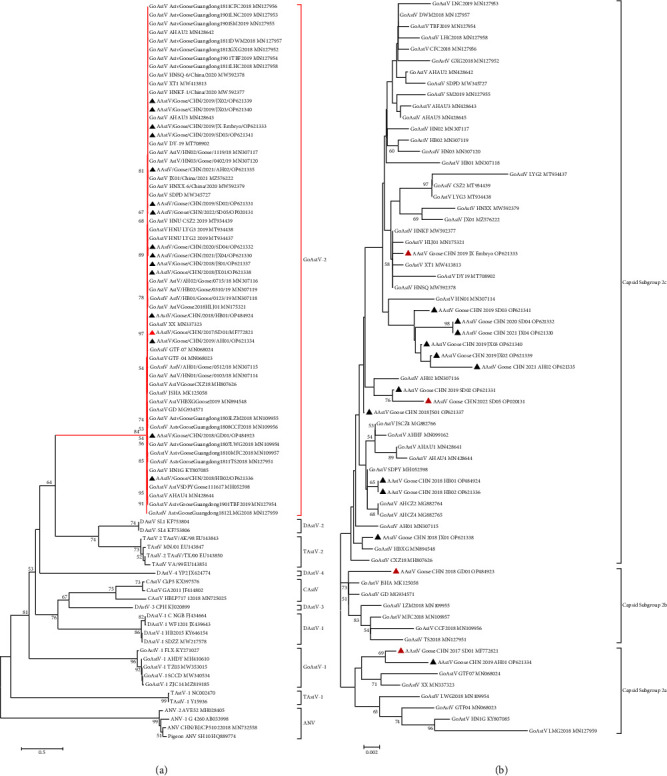
Phylogenetic analysis of isolates in this study. (a) Phylogenetic analysis of the RdRp genes of astroviruses using MEGA 7.0 software. The tree was constructed based on about 1500 nt (nucleotide) sequences using the neighbor-joining method with 1000 bootstrap replicates and the maximum composite likelihood model. Node labels indicated bootstrap values, and bootstrap values <50% were hidden. The strains isolated in this study are indicated by a black triangle and strain SD01 is indicated by a red triangle. (b) Phylogenetic analysis of goose astrovirus genotype G2. Phylogenetic relationship analysis was based on the amino acid sequences of ORF2. The trees were generated using MEGA 7.0 software and the neighbor-joining method with 1000 bootstrap replicates and the evolutionary distances were computed using the JTT matrix-based method. The strains isolated in this study are indicated by a black triangle and four representative isolates are indicated by a red triangle.

Following a previously described method [8], the complete genome sequences of all 15 isolates were obtained and deposited in the GenBank database under the accession numbers indicated in Table 2. All these sequences were aligned with MegAlign, and the overall nucleotide identity between isolates was 97.6% to 99.8%, including the first characterized reference GoAstV isolate SD01. The RdRp gene nucleotide sequences of these isolates were phylogenetically analyzed as shown in Figure 2(a). Together with the majority of the GoAstVs reported previously from China, the 15 GoAstV isolates were assigned to group GoAstV-2.

A second phylogenetic tree was constructed by comparing the deduced amino acid sequences of the full-length ORF2. The distribution of the isolates was consistent with the results obtained by the RdRp gene analysis (Figure 2(b)). Further examination of the GoAstV-2 isolates indicated that the 15 isolates in this study could be assigned to three capsid subgroups (2a, 2b, and 2c). Using the amino acid positions of the reference GoAstV SD01, 28 amino acid substitutions were identified in contrast to reference strain SD01 (Table 3). As such, we mapped the positions of the capsid and 15 substitutions mapped in the capsid spike domain, and five substitutions (E456D, A464N, L540Q, S586T, and D587T) were located within the predicted antigenic epitopes, as described previously [24].

### 3.4. Antigenic Relationship between the GoAstV Isolates

**Figure 3:**
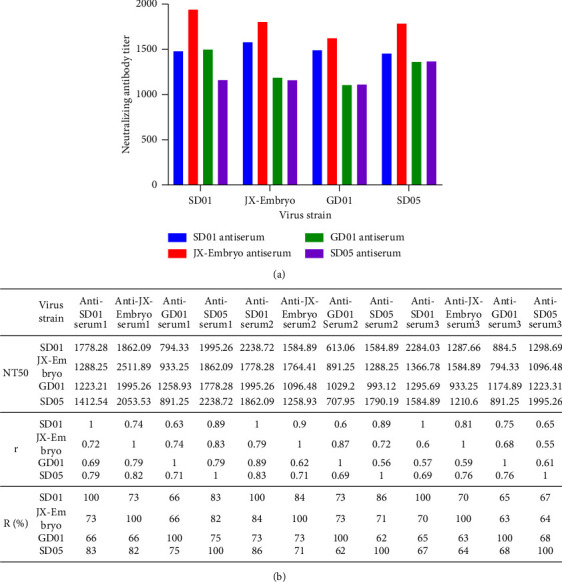
Serum neutralization properties of four GoAstV isolates. (a) Cross-neutralization activity between the SD01 (MF772821), JX-embryo (OP621333), GD01 (OP484923) and SD05 (OP020131) isolates. Antiserum cross-neutralizing antibody titers towards isolates SD01, JX-embryo, GD01 and SD05 were calculated by the reed-muench method. (b) The “NT_50_” “*r*” and “*R*” values between the four isolates.

Because the AstV capsid spikes were predicted to project from the surface of the virion, they are likely to be the major determinant of virus antigenicity [25]. To examine the effects of polymorphic variations on the neutralization sensitivity of the isolates, we selected the isolates JX-embryo, GD01, and SD03 representing the capsid subgroups for antiserum production in healthy geese. Antiserum against reference isolate SD01 was included. As shown in Figure 3(a), antisera effectively neutralized the GoAstV isolates with NT_50_ titers ranging from 1 : 613.06 to 1 : 2511.89. According to Archetti and Horsfall's analysis, the “*R*” values calculated from the heterologous and neutralization titers were between 62% and 86% (Figure 3(b)), indicating that the observed amino acid variations had no significant effect on the neutralizing activities of these GoAstV isolates.

## 4. Discussion

Outbreaks of AstV-induced gout among goslings have been sequentially reported in China and have caused considerable economic losses. We started to investigate the occurrence of natural infections of AstV in commercial flocks after the identification of the etiological relationship between virus infection and fatal gout in goslings [12]. This study has revealed that the newly emerged GoAstV is common in commercial goose flocks based on the following results: (i) viral RNA was detected in both dead-in-shell embryos and day-old hatched goslings, providing evidence for vertical transmission under natural conditions; (ii) detection of neutralizing antibodies in serum samples suggested that commercial geese are commonly exposed to GoAstV; (iii) according to phylogenetic analysis of the genomes of the isolates, the GoAstVs isolated from both goose embryos and clinically dead geese in the present study were clustered together in group GoAstV-2.

The detection and isolation of GoAstV in dead-in-shell embryos indicated that the virus can be transmitted vertically under natural conditions. This is consistent with the detection of AstV infections in chicken, duck, and goose embryos [6]. The vertically transmitted virus may originate from in-lay breeder geese via their eggs, as this was reproduced by experimental infection of WuLong breeder geese [26]. In addition, external egg contamination is a likely source of virus infection because of poor cleaning and insufficient disinfection in the goose hatchery. Consequently, some infected embryos may hatch shedding the virus [27]. This was supported by the detection of virus RNA in the cloacal swabs of day-old hatched goslings. The first-day infected carriers and infective viruses excreted in their feces explain, to some extent, the occurrence of disease outbreaks in goslings as young as 3–5 days in the field [28]. Based on an earlier report, AstV vertical transmission caused not only prehatching mortality of embryos but also runting and stunting in young birds [6]. The potential growth problems induced by GoAstV infection in goslings need to be further investigated.

In this study, GoAstVs were isolated from commercial geese flocks from different goose-rearing regions. This was consistent with other reports that GoAstV is widely distributed in China [29–31]. Genomic sequence analysis revealed that all 15 isolates were assigned to the GoAstV-2 group, but a wide range of amino acid sequence variations were identified in the capsid protein, including residues that were predicted to be associated with the formation of B cell antigenic epitopes [24]. According to the capsid protein sequences, the 15 isolates can be assigned to three capsid subgroups. Previous studies have shown that capsid sequence variation is likely to be reflected in substantial antigenic diversity. We raised antisera against representative isolates of the capsid subgroups, and no significant difference was observed between isolates in cross-neutralization tests. Thus, our results suggest that the observed mutations in the capsid protein do not have the capacity to cause significant antigenic variation.

Serological assays using the neutralization of the virus as a quantitative measurement of antibodies have been used to evaluate the extent of AstV infection among human and animal populations [32, 33]. However, the poor growth of avian AstVs in cell culture has hampered the widespread use of this assay to test field sera. In this study, serial passaging increased the growth of GoAstV in LMH cells [18, 34]. Moreover, the detection of the residual virus by MAb-based IFA significantly modified the determination of the neutralization endpoint because of the slight cytopathic effect of GoAstV in the cell culture. Considering that no approved vaccine is currently available, the finding that the tested serum samples from four flocks displayed seropositivity rates of 41.7% to 61.1% to GoAstV neutralizing antibodies suggested exposure to the virus. The results from the serum samples at slaughter (approximately 120 days), which showed high seropositivity, indicated that infections had persisted in the farm since the first identification of GoAstV. The isolation of GoAstV SD05 from the tissues of 50-day-old geese in the farm also supported the conclusion that AstV contamination of the environment in goose houses may be high. These findings are consistent with the findings regarding AstV infection in chicken and turkey flocks [35, 36]. Further investigations may be required involving in-lay breeder flocks to provide an overview of AstV infection.

In summary, the viral and serological detection of GoAstV in samples of different origins demonstrated that the emerging AstV has spread widely among commercial goose flocks. Infection with this virus causes high mortality in young goslings. The detection of GoAstV from dead-in-shell embryos and day-old goslings supported the view that the virus is vertically transmitted and that the virus may cause hatchability problems. Based on the cross-neutralization results, the viruses belonging to GoAstV-2 did not exhibit substantial antigenic variation, but their antigenic relationship with GoAstV-1 isolates needs to be evaluated.

## Figures and Tables

**Table 1:** Detection of serum neutralizing antibody against GoAstV in goslings.

Collection date	Location	No. tested	No. positive	Positive rate (%)	Antibody titer
May. 2017	Shandong	132	55	41.7	1 : 27–1 : 741
Dec. 2019	Shandong	108	48	44.4	1 : 137–1 : 562
Sept. 2021	Shandong	92	49	53.2	1 : 112–1 : 676
Dec. 2021	Anhui	180	110	61.1	1 : 27–1 : 501

**Table 2:** Information on the isolated strains included in this study.

Strain name	GenBank accession number	Collection date	Host	Province
AAstV/goose/CHN/2017/SD01	MF772821	2017-04-01	Goose	Shandong
AAstV/goose/CHN/2019/SD02	OP621331	2019-05-03	Goose	Shandong
AAstV/goose/CHN/2019/SD03	OP621341	2019-10-09	Goose	Shandong
AAstV/goose/CHN/2020/SD04	OP621332	2020-11-18	Goose	Shandong
AAstV/goose/CHN/2022/SD05	OP020131	2022-01-16	Goose	Shandong
AAstV/goose/CHN/2019/AH01	OP621334	2019-11-15	Goose	Anhui
AAstV/goose/CHN/2021/AH02	OP621335	2021-05-08	Goose	Anhui
AAstV/goose/CHN/2018/GD01	OP484923	2018-03-09	Goose	Guangdong
AAstV/goose/CHN/2018/HB01	OP484924	2018-04-11	Goose	Hebei
AAstV/goose/CHN/2018/HB02	OP621336	2018-04-22	Goose	Hebei
AAstV/goose/CHN/2018/JS01	OP621337	2018-02-06	Goose	Jiangsu
AAstV/goose/CHN/2018/JX01	OP621338	2018-03-29	Goose	Jiangxi
AAstV/goose/CHN/2019/JX02	OP621339	2019-12-02	Goose	Jiangxi
AAstV/goose/CHN/2019/JX03	OP621340	2019-12-02	Goose	Jiangxi
AAstV/goose/CHN/2021/JX04	OP621330	2021-03-16	Goose	Jiangxi
AAstV/goose/CHN/2019/JX-embryo	OP621333	2019/12/16	Goose	Jiangxi

**Table 3:** Summary of the amino acid mutations in the capsid protein for the 15 isolates and strain SD01.

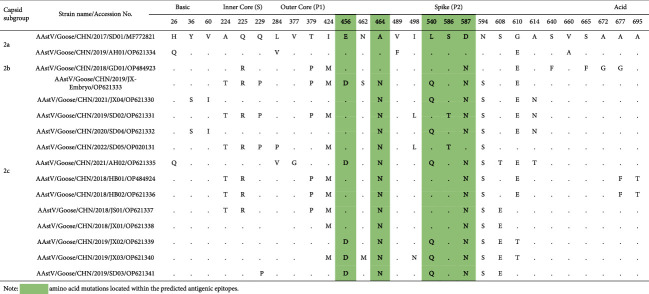

## Data Availability

The data that support the findings of this study are available from the public database GenBank, and the accession numbers are included within the article.
